# Storage temperature and quality dynamics of sun-aged red date vinegar beverage

**DOI:** 10.3389/fnut.2026.1839712

**Published:** 2026-05-18

**Authors:** Zeshan Ali, Zinanone Rosaire Brottier, Jameel M. Al-Khayri, Muhammad Azhar, Othman Al-Dossary, Sam Al-Dalali, Bader Alsubaie, Mustafa I. Almaghasla

**Affiliations:** 1 College of Food Science and Technology, Bohai University, National and Local Joint Engineering Research Center of Storage, Processing and Safety Control Technology for Fresh Agricultural and Aquatic Products, Jinzhou, Liaoning, China; 2 Department of Agricultural Biotechnology, College of Agriculture and Food Sciences, King Faisal University, Al-Ahsa, Saudi Arabia; 3 Department of Applied Data Science, Shue Yan University, Hong Kong, China; 4 Department of Food Science and Technology, Faculty of Agriculture and Food Science, Ibb University, Ibb, Yemen; 5 Plant Pests and Diseases Unit, College of Agriculture and Food Sciences, King Faisal University, Al-Ahsa, Saudi Arabia

**Keywords:** gogi berry juice, machine learning, quality, sensory evaluation, shelf-life prediction, thermal storage, vinegar-based beverages

## Abstract

**Introduction:**

This study investigates the formulation, processing stability, and storage behavior of a vinegar-based beverage derived from red date (*Ziziphus jujuba* Mill.) vinegar produced via controlled fermentation and traditional sun-aging. To improve flavor and reduce sourness, goji berry juice and honey were added to the final formulation.

**Methods:**

The beverage was stored at 25°C, 40°C, and 50°C for 2 months to evaluate its thermal stability. Phytochemical content and antioxidant activity (DPPH, ABTS) were measured. Sensory evaluation and volatile profiling using a 28-sensor electronic nose were performed. Random Forest modeling, hierarchical clustering, feature importance analysis, and kinetic/Arrhenius modeling were applied to assess thermal markers and phenolic degradation.

**Results:**

Elevated temperatures, particularly 50°C led to notable deterioration in phytochemicals, including reductions in total phenolic content and antioxidant activity. Sensory evaluation confirmed optimal acceptability at 25°C, whereas higher storage temperatures caused flavor imbalance and quality deterioration. Volatile profiling revealed temperature-dependent shifts in aroma compounds. The Random Forest model achieved 95% accuracy in classifying storage conditions, and feature analysis and kinetic modeling identified key thermal markers and quantified phenolic degradation.

**Discussion:**

Overall, the findings underscore the importance of controlled storage and demonstrate the potential of multivariate and machine learning tools for shelf-life prediction and real-time quality monitoring in thermosensitive beverages.

## 1 Introduction

Functional beverages enriched with plant-derived bioactive compounds represent complex food matrices whose composition and stability are strongly influenced by formulation and processing conditions. Vinegar-based beverages incorporating fruit-derived components have emerged as technologically relevant systems due to their organic acid profiles, phenolic composition, and volatile interactions, all of which govern sensory performance and storage behavior. These beverages typically contain phenolics, organic acids, and antioxidant compounds that influence chemical stability, physicochemical properties, and product quality during storage (1, 2).

Date vinegar, derived from the fermentation of *Ziziphus jujuba* Mill. (commonly known as jujube or Chinese date), is naturally rich in polyphenols, organic acids, and sugars, which contribute to its antioxidant activity and tangy flavor (3). Likewise, goji berry (*Lycium barbarum* L.) juice is rich in polysaccharides, flavonoids, carotenoids, and phenolics, which are associated with reducing oxidative stress and supporting the immune system (4). Previous studies have demonstrated that combining goji berries with red dates or their derivatives improves sensory quality and enhances consumer acceptance. For example, Goh et al. (5) found that kenaf tea infused with goji and red dates exhibited improved flavor and aroma, while Wang et al. (6) reported enhanced texture and water retention in goat milk cheese with *L. barbarum* and jujube polysaccharides. These findings guided the inclusion of goji juice and sun-aged date vinegar in the current formulation, aiming to optimize both nutritional and sensory appeal.

However, beverages rich in thermosensitive bioactives are prone to degradation during storage. Heat exposure can reduce phenolic, polysaccharide, and antioxidant levels while altering physicochemical parameters, such as pH and total soluble solids (TSS), thereby impacting product quality and acceptability (7).

Thermal stress also affects sensory perception by modifying taste, aroma, texture, and appearance. For instance, higher temperatures can lead to volatile losses in blackcurrant juice, resulting in a significant decrease in the berry-like odor after storage at room temperature (8). To objectively assess aroma changes, electronic nose (E-nose) systems using metal oxide semiconductor (MOS) sensors offer high sensitivity to volatile shifts (9). In this study, electronic nose (E-nose) profiling was coupled with machine learning—Random Forest classification, Confusion Matrix analysis, and Hierarchical Clustering—to classify and interpret temperature-induced changes in key volatiles (alcohols, esters, sulfur compounds), providing a powerful framework for real-time shelf-life prediction and quality monitoring (10). While E-nose systems effectively capture volatile changes, their complex outputs require advanced analytical tools for interpretation. Machine learning enables robust classification of multidimensional sensor data, while kinetic and Arrhenius modeling provide a mechanistic basis for quantifying degradation and predicting shelf-life. The integration of these approaches, therefore, enables a more comprehensive and predictive evaluation of temperature-driven quality changes.

Accordingly, this study evaluated the thermal stability and shelf-life of a vinegar-based beverage formulated with sun-aged red date vinegar, goji berry juice, and honey. In this context, phenolic and antioxidant measurements were employed as technological indicators of compositional stability during storage rather than as predictors of nutritional or health outcomes. Samples were stored at room temperature (RT, 25 °C), 40 °C, and 50 °C for 2 months. Analyses included physicochemical properties (pH, TSS), phytochemical degradation (total phenolics, antioxidant activity via 2,2-diphenyl-1-picrylhydrazyl (DPPH) and 22'-azinobis [3-ethylbenzothiazoline-6-sulfonic acid) (ABTS)], sensory attributes (appearance, taste, aroma, texture, overall acceptability), and intensity profiling (sweetness, sourness, bitterness, fruitiness). Volatile dynamics were assessed using an E-nose and analyzed using machine learning, while Arrhenius modeling estimated the degradation kinetics. These findings offer comprehensive insights into temperature-driven quality changes, supporting improved formulation, storage, and distribution of vinegar-based functional beverages. Notably, previous studies have largely focused on isolated physicochemical or sensory evaluations, with limited integration of volatile profiling, machine learning, and kinetic modeling in vinegar-based systems. This study addresses this gap by combining these approaches to provide a more comprehensive and predictive understanding of temperature-driven quality changes, thereby offering a novel framework for shelf-life assessment and real-time quality monitoring.

## 2 Materials and methods

### 2.1 Ingredients and raw materials

The beverage formulation utilized key ingredients, including red dates, dried goji berries, natural honey, and distilled mineral water. The dates were sourced from a local market in Xi'an, China, while the goji berries were procured from Ningxia Province, known for its high-quality produce. Natural honey was obtained from a cold-processed honey supplier in Jinzhou, China. The chemicals and standards used in the study, including DPPH, methanol, sodium carbonate, Folin–Ciocalteu reagent, sodium hydroxide, ethanol, aluminum chloride, sodium nitrite, gallic acid, Trolox, and ABTS reagent, were of analytical grade and purchased from Shanghai Macklin Biochemical Co., Ltd., China. For the fermentation process, the microbial strains *Saccharomyces cerevisiae* (Angel Yeast Co., Ltd., China) and *Acetobacter spp*. (Baoji Dingli Biotechnology Co., Ltd., China) were employed and chosen for their efficient fermentation capabilities and stable metabolic activity under controlled conditions.

### 2.2 Preparation of sun-aged red date vinegar

Red date vinegar was produced through a sequential fermentation process, as described in our prior study (11). First, sorghum and wheat bran (1:1, w/w) were milled using a 20-mesh sieve, hydrated with water at a 1:3 ratio (w/v), and steamed at 100 °C for 2 h. After cooling to 35–40 °C, pitted red dates were added at a 1:3 ratio (w/w; relative to the steamed cereal substrate), and Daqu was incorporated at 62.5% (w/w; based on the total solid substrate) to initiate fermentation. The mash was inoculated with *Saccharomyces cerevisiae* (0.5%, w/w) for alcoholic fermentation (7–10 days, 25–30 °C), followed by *Acetobacter spp*. (3%, v/v) for acetic acid fermentation (10–14 days, 30–35 °C). The vinegar was filtered and pasteurized (at 85 °C for 10 min) before being transferred to ceramic jars for sun-aging over 4 months at ambient temperatures (25–35 °C). During sun-aging, the jars were exposed to natural sunlight, with daily temperature fluctuations of 25–35 °C and relative humidity of 60–75%. Daytime sunlight intensity typically ranged from 30 to 60 × 103 lx. The jars were loosely covered with breathable cloth to facilitate air exchange while preventing contamination, following traditional sun-aging techniques that contributed to the development of the vinegar's phenolic and organic acid profiles.

### 2.3 Extraction of goji berry juice

Dried goji berries (50 g) were rinsed and soaked in clean water for 30 min to soften. After draining, the softened berries were blended with 1 liter of mineral water, which served as a clean, standardized extraction medium, until a smooth slurry formed. The mixture was then strained to extract the juice, yielding approximately 150–180 ml, which was used for preparing the beverage samples. To preserve the native phytochemicals and volatile compounds, the juice was neither fermented nor heated.

### 2.4 Beverage formulation and sample coding

Six different beverage formulations were designed as part of a comparative formulation experiment to evaluate how varying ingredient ratios influence physicochemical, phytochemical, sensory, and storage stability. Each formulation represented a treatment level within this design rather than a traditional control-based experiment. Beverage samples were prepared by blending goji berry juice into the sun-aged red date vinegar base, along with honey and water, to produce 100 ml samples. The ingredient ratios varied as follows (red date vinegar/goji berry juice/honey/water, ml/ml/g/ml): A (6/4/4/86), B (7/5/5/83), C (8/6/5/81), D (10/8/6/76), E (12/10/6/72), and F (14/10/9/67). Ingredient ratios were optimized in preliminary trials to ensure that vinegar exceeded goji juice in all formulations, thereby preserving the vinegar-based identity. Honey's balanced sweetness and acidity, combined with varying amounts of vinegar and goji juice, created a gradient in acidity, sweetness, and color for sensory evaluation. Goji berry juice was added post-fermentation to enhance sweetness, aroma, and color, while minimizing phytochemical loss. The final formulation was optimized through sensory panel evaluations, focusing on palatability and acceptability (12, 13).

#### 2.4.1 Thermal storage testing

Samples were stored at RT (25 °C), 40 °C, and 50 °C for 2 months to simulate the effects of thermal exposure on the beverage. While 50 °C is unlikely to occur under typical shelf conditions, it was included as an accelerated-stability testing temperature to evaluate thermal degradation behavior and predict shelf-life trends within a shorter experimental period. Such conditions are commonly used to simulate cumulative thermal stress that may arise during transportation or temporary storage in warmer climates. All samples were stored in light-protected containers to prevent oxidation and photodegradation. Although these extreme temperatures do not reflect typical long-term storage conditions, they provide valuable insights into the potential effects of transient heat exposure on product stability.

### 2.5 Physical-chemical analysis

The pH of the beverage samples was determined using a pH meter (InoLab WTW Series 730, China) with a glass electrode. Before measurement, the pH meter was calibrated using standard buffer solutions across the expected pH range, according to the manufacturer's guidelines. All pH measurements were taken at 25 °C. TSS, expressed as °Brix (grams of sucrose equivalent per 100 g of solution), was measured using a portable refractometer (Shanghai Lichen Bangxi Instrument Technology Co., Ltd). Calibration was conducted using sucrose solutions of known concentrations, resulting in a coefficient of determination of *R*^2^ = 0.98. Each measurement was repeated in triplicate to ensure accuracy.

### 2.6 Phytochemical analysis

Total phenolic content (TPC) was determined using the Folin–Ciocalteu method (14). Quantification was performed using standard calibration curves prepared from gallic acid solutions, which showed strong linearity (*R*^2^ ≥ 0.99), and results were expressed as gallic acid equivalents (GAE). Antioxidant activity was assessed using DPPH and ABTS assays (15, 16). Trolox calibration curves demonstrated high linearity (*R*^2^ ≥ 0.99), and results were expressed as Trolox equivalents (TE). All measurements were performed in triplicate.

### 2.7 Sensory and flavor analyses

#### 2.7.1 Ethical assessment and consent

The sensory evaluation in this study was conducted with adult human volunteers, following ethical guidelines for sensory research. Since the study involved low-risk, commercially available food products (red date vinegar and goji berry juice), which are commonly consumed in China, and did not involve the collection of personal or sensitive data, formal ethics committee approval was not required. However, all participants were fully informed about the study's purpose, procedures, and their rights, including the ability to decline participation or withdraw at any time without penalty. Written informed consent was obtained from all participants, and no vulnerable populations were included. Confidentiality was maintained throughout the study. The research adhered to the ethical standards outlined in the Declaration of Helsinki and the general principles for human sensory and consumer testing (17).

#### 2.7.2 Sensory evaluation

Sensory evaluation was conducted with 30 trained panelists (16 males and 14 females), selected to match the typical consumer demographic for sun-aged red date vinegar-based beverages. The selection criteria included an age range (e.g., 18–45 years) and consumption habits, such as familiarity with health beverages or fermented drinks, to ensure that the panel represented individuals likely to consume the product. The panelists were pre-screened and trained to assess key sensory attributes, including sweetness, sourness, bitterness, fruity notes, aroma, and mouthfeel. The training procedure consisted of a two-day session where panelists were introduced to the sensory attributes, familiarized with the rating scales, and calibrated using standard reference samples. During training, panelists practiced assessing different levels of intensity for each attribute to ensure consistency. The evaluation was performed in two phases: first, hedonic sensory attributes were assessed, where panelists evaluated appearance, taste, texture, aroma, and overall acceptability using a 9-point hedonic scale (1 = dislike extremely to 9 = like extremely) (18). In the second phase, intensity-based sensory attributes were rated by the same panelists, who assessed their intensity using a 5-point scale (0 = not perceivable to 5 = extremely strong) for attributes such as sweetness, sourness, bitterness, and fruitiness. Panel performance was monitored throughout the evaluation by conducting periodic checks to ensure consistency in attribute ratings.

The beverage samples were stored at 25 °C, 40 °C, and 50 °C for 2 months prior to sensory evaluation. After storage, the samples were blind-coded and presented in random order across multiple sessions. Freshly prepared samples were included as reference controls and were prepared daily during the seven-day evaluation period to ensure consistency. All evaluations were conducted in a controlled sensory laboratory with standardized lighting and temperature conditions. Hedonic scores and intensity ratings were analyzed to assess overall acceptability and changes in sensory profile associated with storage conditions (19).

### 2.8 Electronic nose analysis

The E-nose protocol was adapted from established methods with minor modifications (20). Volatile profiles of beverage samples stored at 25 °C, 40 °C, and 50 °C for 2 months were analyzed using 28 metal oxide semiconductor sensors (S1–S28), each selectively responsive to different classes of aroma compounds. Measurements were conducted with a 1-min sample injection at room temperature, at a flow rate of 1 L/min, followed by a 20-min cleaning cycle to prevent sensor carryover. Radar plots were generated to visualize sensor responses and assess temperature-induced differences in volatile patterns. Sensor detection ranges and compound groupings are summarized in Supplementary Table S1. Each sample was measured in triplicate to ensure data consistency and reliability. The analysis focused on pattern recognition and relative classification of volatile profiles, suitable for exploratory and multivariate assessments.

### 2.9 Machine learning-based volatile classification

E-nose sensor data were obtained from repeated measurements of six beverage formulations (A–F) stored at 25 °C, 40 °C, and 50 °C for 2 months. These data, comprising multiple replicate readings per sample across 28 MOS sensor features, were preprocessed through normalization, feature scaling, and imputation of missing values to ensure consistency. A Random Forest classifier was trained on 70% of the dataset and tested on the remaining 30% to model the relationship between volatile profiles and storage temperatures. The model utilized 100 decision trees, employing Gini impurity as the split criterion and a fixed random seed [42] for reproducibility. Model performance was evaluated using a confusion matrix. Hierarchical clustering was used to visualize similarities in sensor responses across temperature groups, and feature importance analysis was applied to identify the most discriminative sensors. All analyses were performed using Python (v3.10) and Scikit-learn (v1.2.2) within the Google Colab environment.

### 2.10 Kinetic modeling and arrhenius analysis

First-order degradation kinetics were used to model the thermal degradation of TPC, based on measurements collected at 0, 1, and 2 months at three storage temperatures (21). Degradation rate constants (k) were calculated using the equation below:


$$K=\frac{\ln(C_{0}/C_{t}\;)}{t}$$ (1)

Where C_0_ and C_t_ denote the average TPC at time 0 and time t (in months), respectively, the natural logarithm of the rate constants (ln k) was plotted against the reciprocal of absolute temperature (1/T, in K^−1^) to generate the Arrhenius plot. The activation energy (Ea, kJ/mol) was calculated from the slope of the regression line using the Arrhenius equation:


$$\begin{array}{l} \ln\left(k\right)=\ln\left(A\right)-\frac{E_{a}}{R}\cdot \;\frac{1}{T} \end{array}$$ (2)

where *R* = 8.314 J·mol^−1^·K^−1^. All calculations were based on triplicate TPC measurements. Data analysis and linear regression were conducted using Python (version 3.10) with the SciPy and Matplotlib libraries. Full raw data and calculated kinetic parameters are provided in Supplementary Table S2.

### 2.11 Statistical analysis and visualization

All results were expressed as mean values. One-way ANOVA followed by Tukey's test (*p* < 0.05) was used to assess differences in pH and TSS over storage. Sensory data were visualized using radar charts to compare attributes at different temperatures. E-nose responses from 28 sensors were plotted as radar diagrams to visualize relative aroma profiles across treatments; data interpretation was descriptive, based on compound-specific sensor sensitivities. First-order kinetic modeling and Arrhenius plots were used to estimate reaction rates and the activation energy in phenolic degradation. Machine learning-based analysis of E-nose data, including classification, clustering, and feature importance, was conducted as described in Section 2.9. Data analysis and visualization were performed using Microsoft Excel (2016), SPSS (version 29.0.2.0), and OriginPro (2021).

## 3 Results and discussion

Storage temperature significantly influenced compositional stability, with higher temperatures accelerating phenolic degradation, reducing antioxidant capacity, altering volatile composition, and lowering sensory scores. E-nose analysis detected temperature-dependent shifts in aroma-related compounds, while machine learning models successfully classified storage conditions and identified the most thermally responsive sensors. Shelf-life behavior was further characterized using first-order kinetics and Arrhenius modeling of phenolic degradation patterns, confirming temperature-driven deterioration during storage.

### 3.1 . Changes in pH and TSS during storage

**Figure 1:**
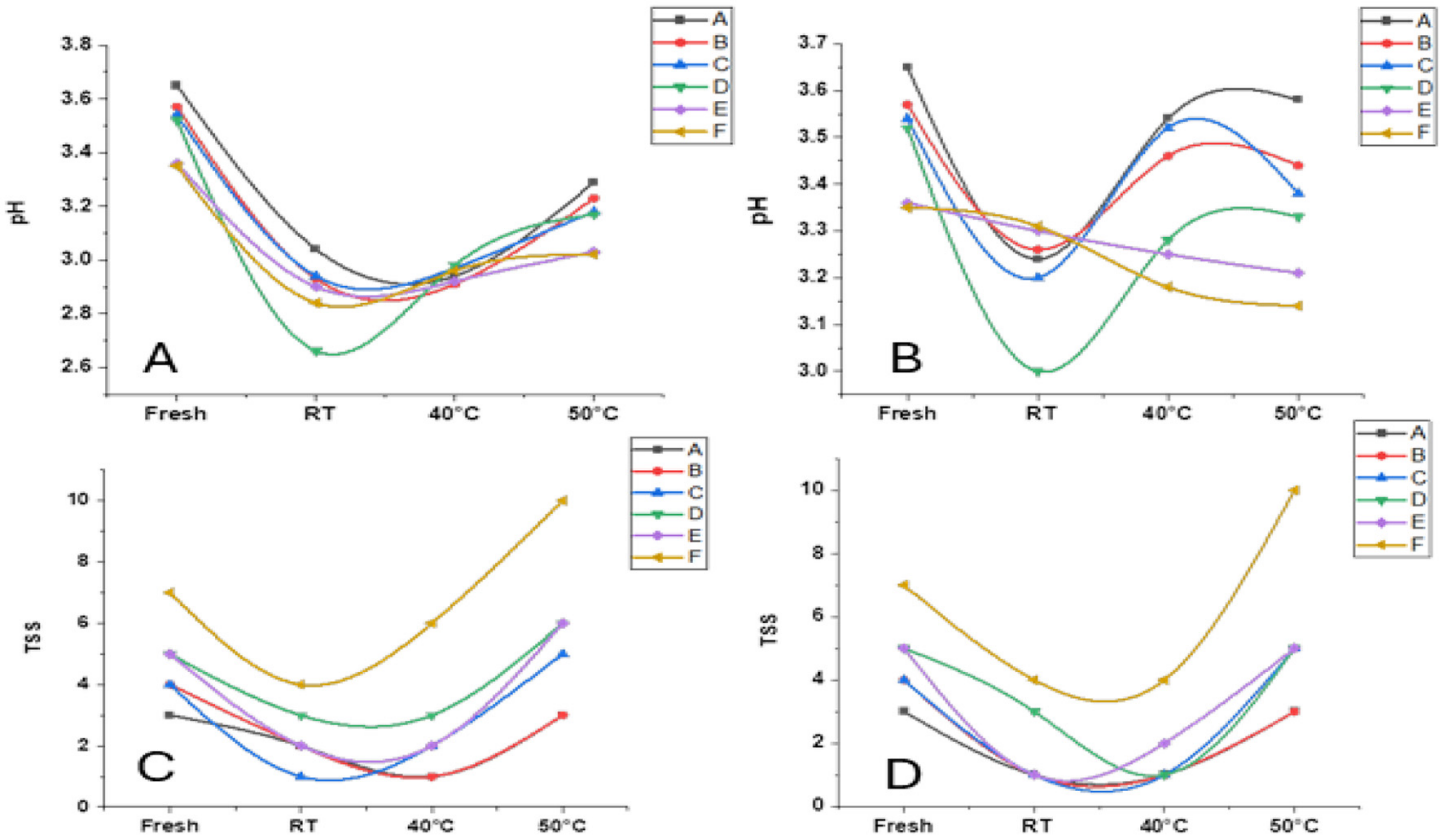
Changes in pH **(A, B)** and total soluble solids (TSS) **(C, D)** of functional beverage samples **(A–F)** during storage at different temperatures. Measurements were taken for fresh samples and after storage at room temperature (RT), 40 °C, and 50 °C for 1 month **(A, C)** and 2 months **(B, D)**. Data represent mean values of triplicate analyses.

Figure 1 illustrates changes in pH and TSS of beverage samples **(A–F)** after 1 and 2 months of storage at RT, 40 °C, and 50 °C. After 1 month, significant pH declines were observed, particularly at RT, where Sample D dropped below 2.9. Interestingly, Samples A, B, and C exhibited partial pH recovery at elevated temperatures, likely due to the thermal inactivation of acid-producing microbes or degradation of organic acids. By the second month, this trend became more pronounced: Samples A and C showed an increase in pH at 50 °C, likely due to alkaline byproducts or buffering effects from thermal degradation. On the other hand, Sample F showed a continuous decline in pH across all storage conditions, suggesting ongoing acidification driven by heat-stable processes. These findings align with previous studies showing that moderate temperatures promote acidification, while higher temperatures may stabilize pH, depending on microbial activity and buffering capacity (22, 23).

TSS followed a different trend. A general decline was observed at RT and 40 °C after 1 month, followed by a sharp increase at 50 °C after 2 months (Figures 1C, D). Sample F consistently exhibited the highest TSS (~10.2 ° Brix), while sample A recorded the lowest, particularly at RT and 40 °C. The rise of TSS at higher temperatures is likely linked to the thermal degradation of polysaccharides and the release of soluble sugars, as noted in heat-treated fruit pulps (24). Statistically significant differences (*p* < 0.05), indicated by superscript letters, highlight the combined effect of formulation and temperature on sugar stability and concentration.

### 3.2 Phytochemical changes during storage

#### 3.2.1 Degradation of TPC

**Figure 2:**
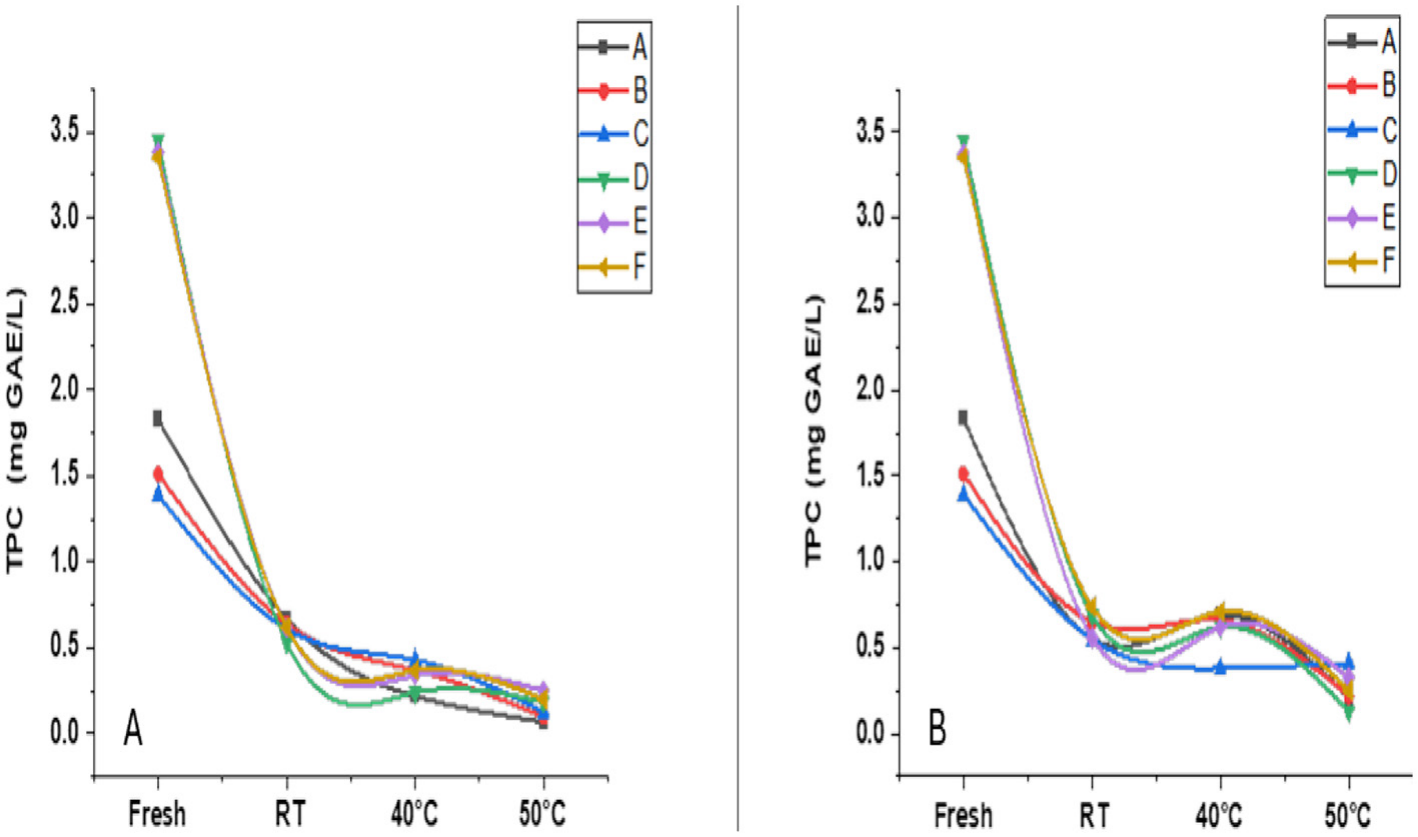
Total phenolic content (TPC) of functional beverage samples **(A–F)** stored at room temperature (RT), 40 °C, and 50 °C, compared to fresh samples. **(A)** shows TPC changes after 1 month of storage; **(B)** shows changes after 2 months. Values are expressed as mg gallic acid equivalents per liter (mg GAE/L). All measurements were performed in triplicate.

Figure 2 displays the TPC of beverage samples stored under different conditions. Fresh samples had high TPC levels, with sample F recording the highest (~3.6 mg GAE/ml). After 1 month, a marked decline was observed in all samples, especially under higher temperatures (Figure 2A). By the end of 2 months, all samples had TPC levels below 0.5 mg GAE/ml at 50 °C (Figure 2B). These reductions are attributed to thermal degradation and oxidation of phenolics, consistent with observations in other fruit-based juices, such as *Phyllanthus emblica*, where elevated storage temperatures led to significant phenolic losses (25). Similarly, in lingonberry juice, anthocyanins, a key class of phenolics, were the least stable under thermal treatment, with half-lives significantly reduced at higher temperatures (26). Significant differences (*p* < 0.05) among formulations and storage conditions indicate varying phenolic stability. These results highlight the need for optimized storage protocols to retain phenolics, which are vital for beverage antioxidants and functional properties.

#### 3.2.2 Antioxidant activity under thermal and storage stress

**Figure 3:**
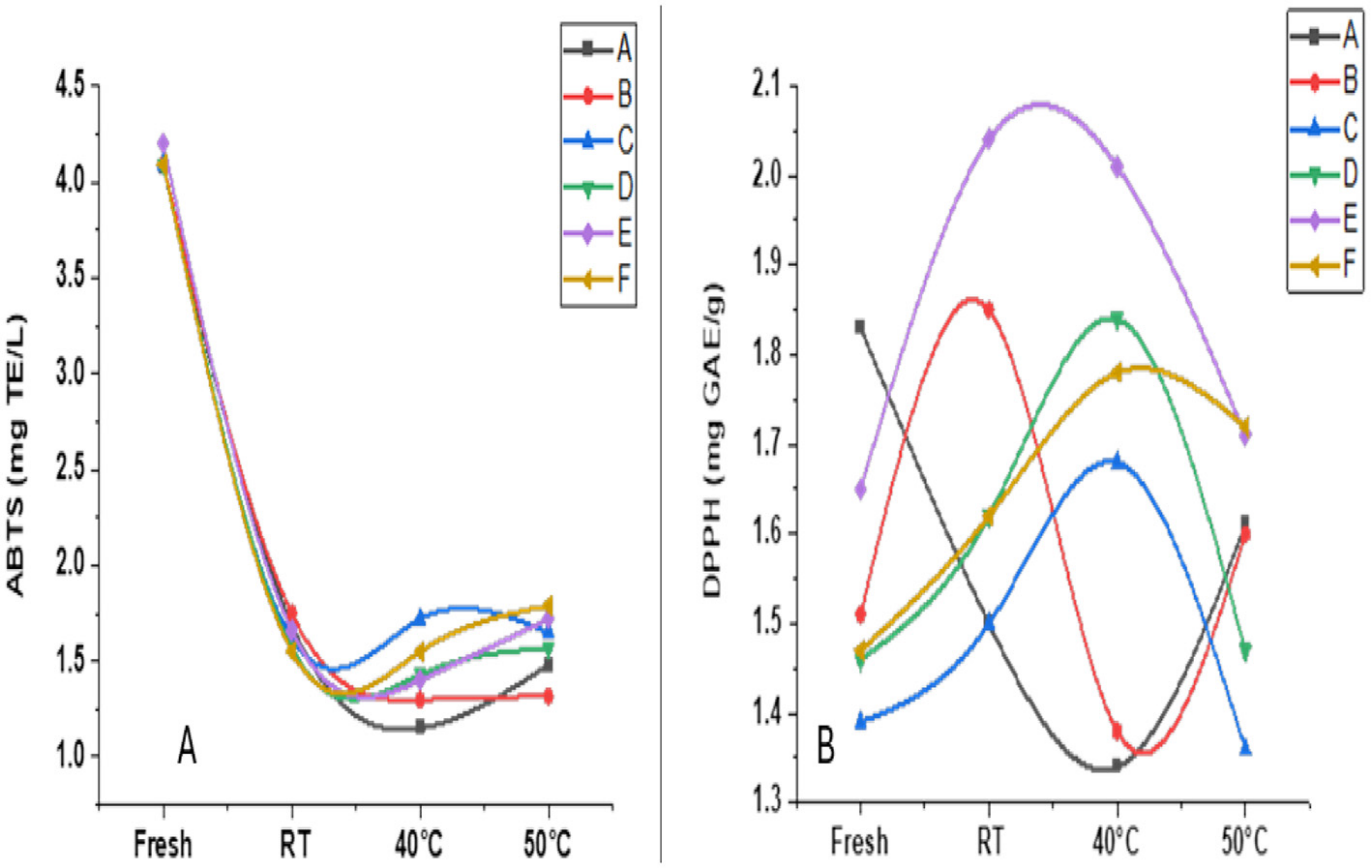
Antioxidant activity of functional beverage samples **(A–F)** assessed by ABTS **(A)** and DPPH **(B)** radical-scavenging assays after 2 months of storage at room temperature (RT), 40 °C, and 50 °C, compared to fresh samples. Results are expressed as mg Trolox equivalents per liter (mg TE/L) for ABTS and mg gallic acid equivalents per gram (mg GAE/g) for DPPH. All measurements were performed in triplicate.

Figure 3 illustrates the antioxidant activity of beverage samples after 2 months, as assessed using the ABTS (Figure 3A) and DPPH (Figure 3B) assays. ABTS activity declined significantly from ~4.2 mg TE/ml in fresh samples to 1.2–1.7 mg TE/ml, particularly at RT and 40 °C, reflecting the degradation of thermolabile antioxidants under oxidative and thermal stress.

In contrast, DPPH activity displayed a non-linear trend. Sample E showed an unexpected increase at RT (~2.1 mg GAE/g), followed by a decline at higher temperatures. This may indicate the transient formation of antioxidant intermediates or differences in assay sensitivity.

Significant differences (*p* < 0.05) across formulations highlight the influence of matrix composition and storage conditions on antioxidant retention. Similar trends have been observed in thermally processed juices and nutraceutical blends, where antioxidant losses were linked to phenolic degradation and structural changes during storage (27, 28). Overall, these findings emphasize the complexity of antioxidant behavior and support the need for formulation-specific strategies and multi-assay approaches to maintain functional quality during storage.

### 3.3 Sensory quality and intensity attributes

#### 3.3.1 Hedonic sensory attributes

**Figure 4:**
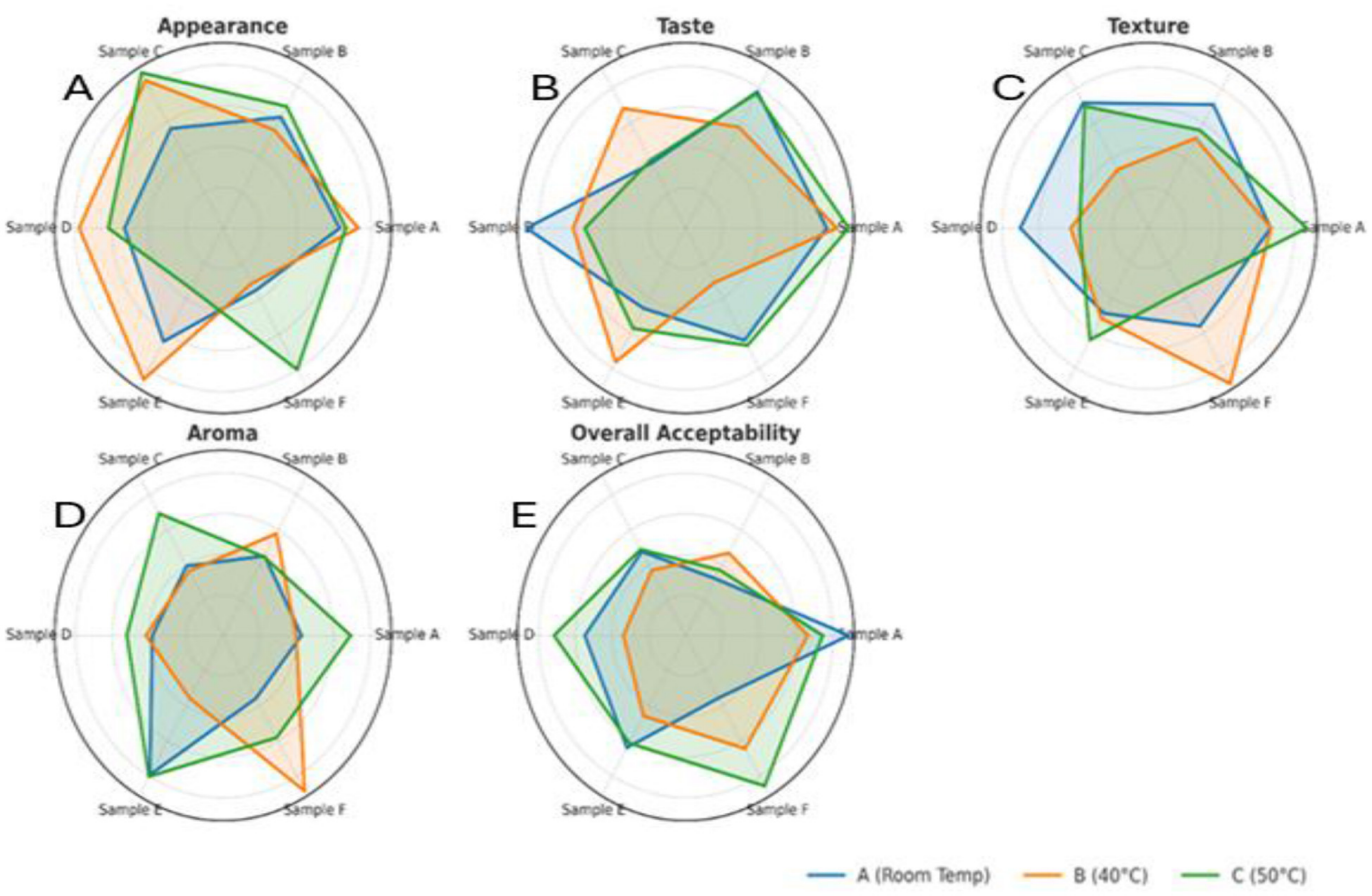
**(A–E)** Radar plots showing hedonic sensory evaluation of functional beverage samples **(A–F)** stored for 2 months at room temperature (blue), 40 °C (orange), and 50 °C (green): **(a)** Appearance; **(b)** Taste; **(c)** Texture; **(d)** Aroma; and **(e)** Overall acceptability.

**Figure 5:**
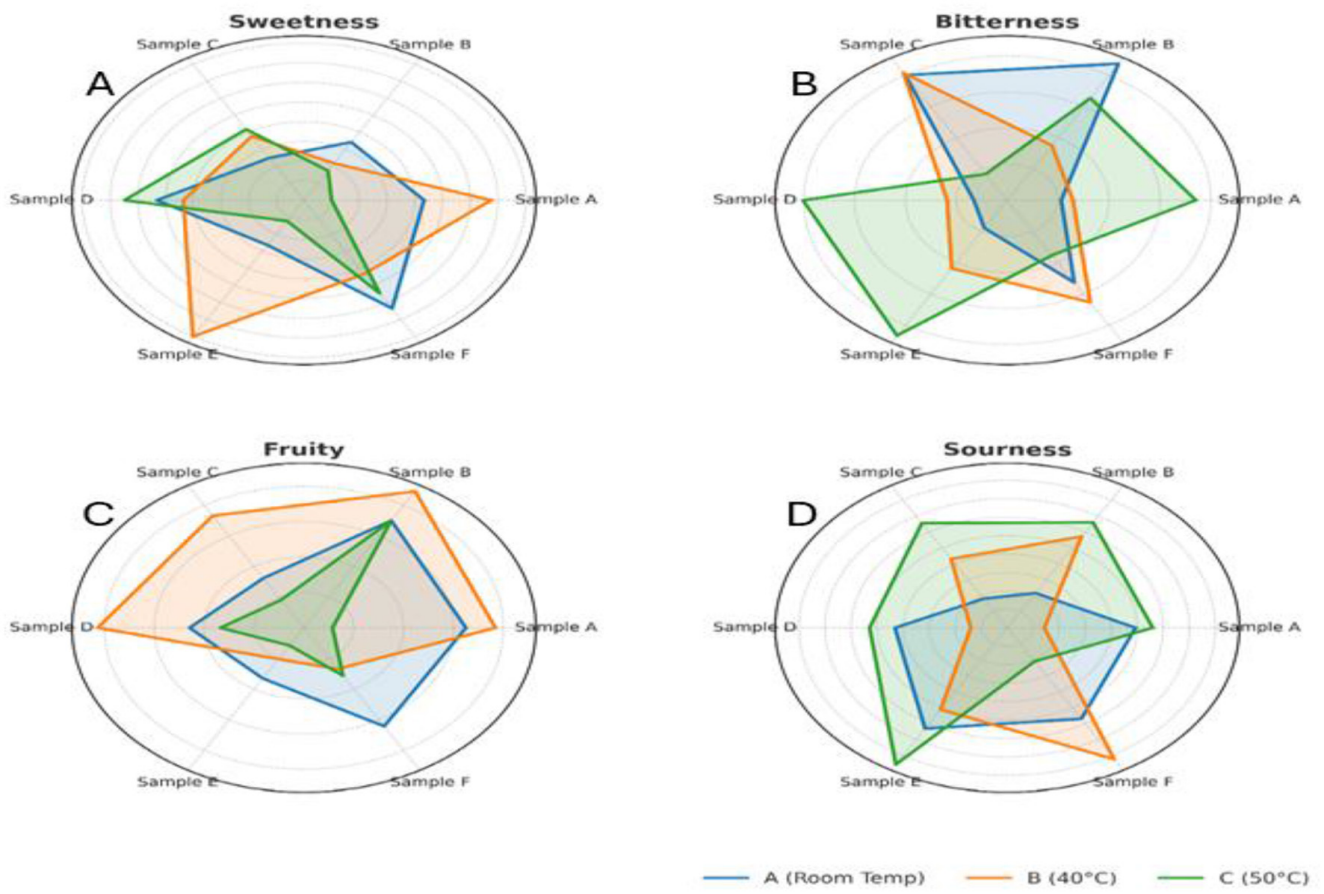
**(A–D)** Radar plots illustrating intensity-based sensory profiling of functional beverage samples **(A–F)** stored for 2 months at room temperature (blue), 40 °C (orange), and 50 °C (green): **(a)** Sweetness; **(b)** Bitterness; **(c)** Fruitiness; and **(d)** Sourness.

The sensory quality of the beverage was evaluated after 2 months of storage at room temperature (40 °C and 50 °C). A dual approach involving hedonic evaluation—encompassing appearance, taste, texture, aroma, and overall acceptability (Figures 4A–E)—and intensity-based sensory profiling (Figures 5A–D) was employed to assess changes in consumer perception and product characteristics.

Samples stored at room temperature consistently received higher hedonic scores across all attributes. Visual quality, especially in samples C and D, remained stable, with good clarity and color retention—key indicators of freshness and appeal (Figure 4A). In contrast, samples stored at 50 °C exhibited visible deterioration, including increased turbidity and browning, indicating reduced visual quality under elevated temperature conditions.

Taste acceptability was also highest at room temperature and 40 °C (Figure 4B). Sample A achieved the highest ratings, likely due to a favorable sugar-to-acid balance and enhanced flavor release from mild thermal exposure. Conversely, beverages stored at 50 °C exhibited notable declines in sweetness, accompanied by the appearance of bitter or off-flavors, which may be attributed to sugar degradation and the formation of bitter metabolites. A similar impact of formulation on taste was demonstrated by Maulida et al. (29), who observed sensory differences driven by ingredient composition in cascara-roselle-red ginger drinks.

Textural perception followed comparable patterns (Figure 4C). Samples stored at lower temperatures maintained desirable viscosity and mouthfeel. Notably, Sample F showed improved texture at 50 °C, potentially due to fiber gelation, honey-induced thickening, or structural matrix changes. In contrast, samples D and E, stored at high temperatures, exhibited sedimentation and texture loss, likely due to fiber breakdown and destabilization of polysaccharides.

Aroma intensity was best preserved in samples stored at RT and 40 °C, particularly for Sample A (Figure 4D). This may be attributed to the release of volatile compounds during mild heating. At 50 °C, significant aroma losses were observed, likely due to volatilization or degradation of aromatic esters and aldehydes. Rezazadeh and Ghasempour (30) emphasized that matrix composition and potential encapsulation mechanisms can help preserve volatiles under thermal stress, which may explain why Sample F retained its aroma despite high-temperature exposure.

Overall, acceptability mirrored these trends (Figure 4E). Samples stored at RT and 40 °C were well received, with Sample A rated highest across categories. Despite the general decline at 50 °C, Sample F maintained relatively high acceptability, indicating greater thermal stability. These results align with those of Laaksonen et al. (31), who demonstrated that the preservation of sensory attributes in blackcurrant juice during storage was influenced by processing methods and formulation components, with specific treatments maintaining better visual and textural qualities over time.

#### 3.3.2 Intensity-based sensory attributes

Quantitative profiling of sweetness, bitterness, fruitiness, and sourness provided deeper insights into the sensory responses influenced by storage temperature. The sweetness was best retained at room temperature (Figure 5A), with samples B and F showing notably higher intensities. At 50 °C, sweetness became inconsistent across samples; however, sample E retained the highest sweetness, potentially due to matrix-specific sugar release or concentration effects during heating. These findings align with those of Kishore et al. (32), who reported that sweetness in purple passion fruit juice increased during early storage at 25 °C. Still, they were better preserved at 8 °C over extended periods, with reduced off-flavor development.

Bitterness increased with temperature (Figure 5B), minimal at RT but intensifying at 40 °C and peaking at 50 °C, especially in samples A, D, and E. This trend is likely linked to the thermal oxidation of polyphenols or the hydrolysis of bitter glycosides under heat. Conversely, samples B and F maintained lower bitterness, possibly due to formulation-specific masking effects or phenolic stability.

Fruitiness peaked at 40 °C (Figure 5C), particularly in samples B, C, and D, suggesting enhanced volatilization of aroma-active compounds under moderate thermal stress. However, a notable decline at 50 °C suggests thermal degradation of volatiles and suppressive interactions between acids and phenolics. Sourness remained stable at room temperature but increased at higher temperatures (Figure 5D), particularly in samples B, E, and F, likely due to the accumulation of organic acids or an altered sweetness–sourness balance.

These trends are supported by recent findings that thermal processing can compromise sensory quality by degrading key flavor compounds and enhancing bitterness. In contrast, non-thermal approaches, such as pulsed electric fields and thermosensation, have demonstrated improved retention of sweetness and fruitiness during storage, highlighting the importance of processing and storage temperature in maintaining sensory integrity (33).

These results indicate that storage at 40 °C may enhance certain sensory traits—particularly sweetness and fruitiness—without substantially increasing bitterness or sourness. In contrast, 50 °C storage induces undesirable shifts in flavor balance, except in specific cases such as Sample E (sweetness preservation) and Sample F (bitterness control). These findings underscore the importance of combining hedonic and intensity-based assessments to guide thermal stability and shelf-life strategies for functional beverages rich in thermosensitive bioactives and acids.

### 3.4 E-nose analysis of volatile aroma patterns

**Figure 6:**
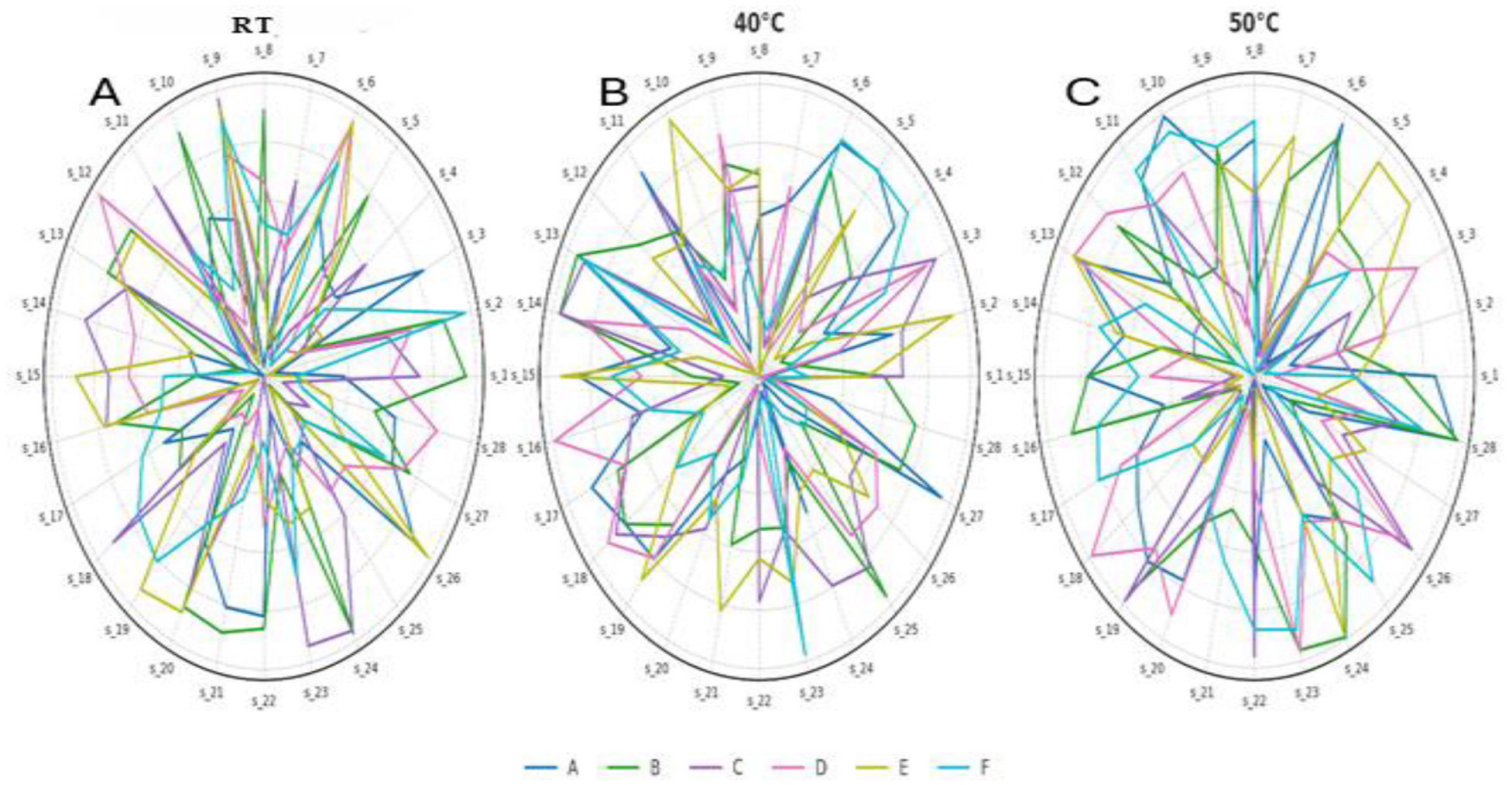
Radar plots of E-nose sensor response patterns for beverage samples **(A–F)** after 2 months of storage: **(A)** room temperature (25 °C), **(B)** 40 °C, **(C)** 50 °C. Each plot shows signal intensities from 28 sensors (S1–S28), highlighting temperature-induced changes in volatile aroma patterns.

Figures 6A–C present a comparative overview of E-nose sensor response patterns for beverage samples (A–F) after 2 months of storage at room temperature (20 °C), 40 °C, and 50 °C. Radar plots illustrate signal intensities across 28 sensors (S1–S28) and capture temperature-induced variations in volatile aroma patterns.

At 25 °C, sensor responses were relatively stable and consistent across all formulations, indicating minimal fluctuation in aroma-active components. Moderate and uniform activity in sensors S2 (alcohols and aldehydes), S8 (short-chain alkanes), S10 (hydrogen), and S24 (alkanes and olefins) indicated the preservation of key volatile constituents, which corresponded with favorable aroma acceptability scores in sensory evaluation.

Storage at 40 °C induced noticeable shifts in sensor response patterns, particularly in S4 (sulfides), S11 (biogas, short-chain alkanes), and S22 (aliphatic hydrocarbons). These changes may reflect partial volatilization or transformation of thermosensitive compounds. Samples B and C exhibited pronounced sensor activity, possibly reflecting enhanced release of volatile compounds such as esters and oxygenated volatiles (e.g., indicated by S13 and S26), which aligns with the improved aroma scores observed at this temperature. In contrast, storage at 50 °C led to more erratic sensor responses. Intensified activity in S1 (alkanes), S7 (natural gas), S13 (ketones and alcohols), and S21 (methanol, amines, and sulfur-related volatiles), alongside suppression in S5 (ammoniates) and S18 (butane), indicated substantial aroma deterioration and potential formation of off-flavor volatiles. These patterns suggest oxidative or thermal breakdown of aromatic constituents, consistent with decreased sensory scores for aroma acceptability at this elevated temperature.

Overall, the radar plots offered clear visual differentiation of aroma stability across storage conditions. The data indicate that moderate storage temperatures (≤ 40 °C) help maintain the integrity of volatile aromas, whereas elevated temperatures accelerate their degradation. These observations align with prior studies on the thermal sensitivity of aroma-active compounds in functional beverages. Mamat et al. (20) demonstrated that an electronic nose with metal oxide sensors can reliably classify juice aroma profiles with high repeatability and sensitivity. Furthermore, Fei et al. (34) confirmed that thermally induced transformations in volatile compounds, such as aldehydes, ketones, esters, and furans, can be effectively detected using E-nose sensor patterns and validated by high-resolution gas chromatography-mass spectrometry (HR-GC-MS) analysis. Their results are consistent with the erratic sensor responses observed at 50 °C in the present study, supporting the interpretation that aroma deterioration occurs via oxidation or thermal degradation pathways. In line with the exploratory objectives of this study, the interpretation of E-nose data focused on comparative visualization rather than on inferential statistical testing of individual sensor outputs. Combined with sensory results, the E-nose analysis highlights the importance of maintaining controlled temperature conditions to preserve the volatile and sensory integrity of thermosensitive bioactive beverages.

While radar plots visually capture temperature-induced shifts in volatile profiles, they offer limited quantitative insight into classification accuracy or sensor contributions. To overcome this, machine learning models—including Random Forest, confusion matrix analysis, and feature importance—were applied to provide a more robust assessment of sensor response patterns across storage conditions. The following sections present these model-driven insights into aroma variation.

#### 3.4.1 Confusion matrix analysis

**Figure 7:**
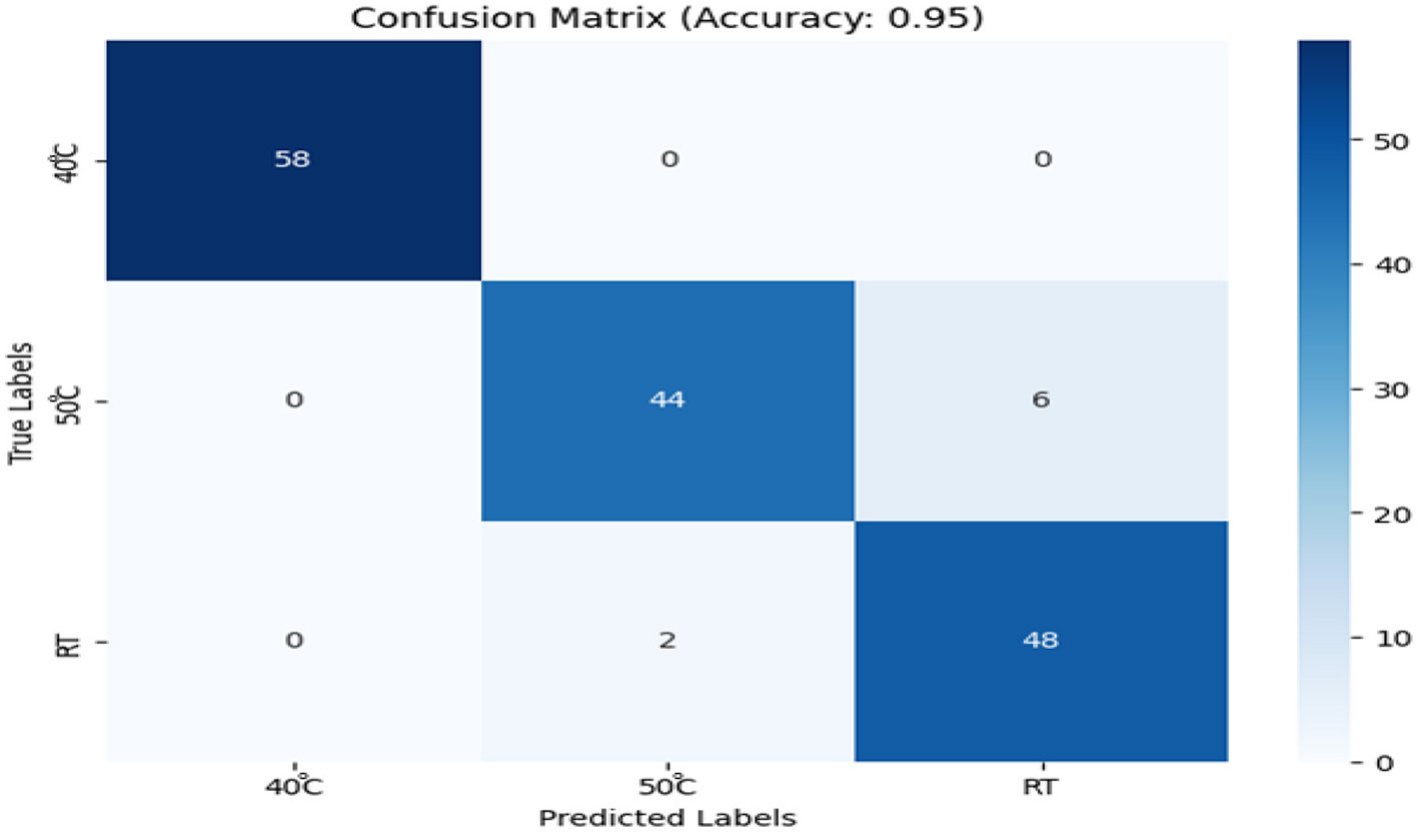
Confusion matrix of random forest classification of storage temperatures using E-nose data (accuracy: 95%).

The confusion matrix (Figure 7) showed that the Random Forest classifier achieved 95% accuracy in classifying temperature conditions. A few misclassifications were observed between the 50 °C and RT groups. These misclassifications were attributed to overlapping volatile profiles, primarily due to oxidative reactions and the formation of volatile esters and alcohols under thermal stress. These compounds, crucial components of the aroma profile, undergo significant changes with temperature, leading to similar sensor responses at 50 °C and RT. Sensors sensitive to alcohols and esters (e.g., Sensors 15 and 1) likely detected these overlapping signatures, resulting in misclassifications.

While RT samples showed moderate sensor responses, the 50 °C samples exhibited higher levels of oxidative byproducts and esterification products, which influenced the sensors' ability to differentiate between the temperature conditions. The overlap of these compounds, particularly alcohols and esters, made it difficult to distinguish subtle temperature-induced changes. Notably, sensors 15 and 1 demonstrated stability in detecting these compounds across RT and 40 °C, while their response at 50 °C became more pronounced due to esterification and oxidation.

Similar applications of Random Forest classifiers in E-nose systems have shown strong performance in discriminating complex volatile profiles. For example, Li et al. (35) reported high classification accuracy in identifying Chinese liquor flavors using a QCM-based E-nose with a Random Forest model, outperforming other algorithms such as LDA and SVM. Likewise, Ni et al. (36) achieved 96% accuracy using a hybrid XGBoost–Random Forest framework for classifying volatile organic compounds, further confirming the utility of ensemble learning in handling overlapping VOC signals in gas sensing systems.

While the confusion matrix confirmed the Random Forest model's classification performance, hierarchical clustering was applied to explore sensor-level response patterns and reveal how specific sensors react to temperature-driven changes in volatile compounds.

#### 3.4.2 Hierarchical clustering (Dendrogram)

**Figure 8:**
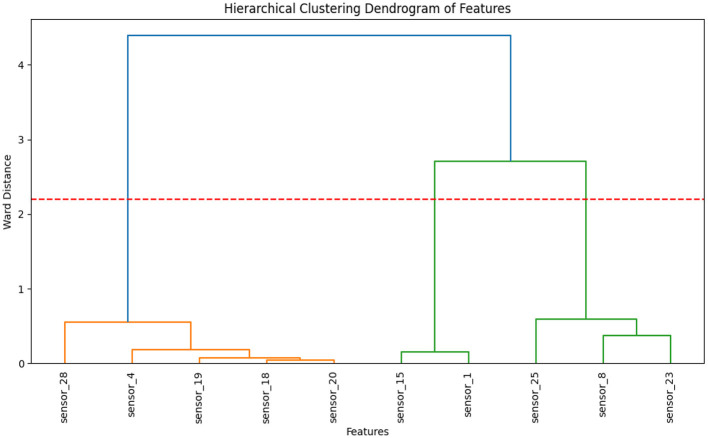
A hierarchical clustering dendrogram of E-nose sensor features, based on the ward distance, shows two main sensor clusters.

The Dendrogram (Figure 8) revealed two distinct clusters of sensors based on their responses to volatile compounds. Cluster 1, consisting of Sensors 28, 4, 19, 18, and 20, primarily responded to alcohols and esters. These sensors showed consistent stability across all temperature conditions, with stable responses to alcohols and esters at RT and 40 °C, indicating their reliability in capturing oxidative byproducts under mild heating. However, at 50 °C, their responses increased significantly due to the formation of alcohols and esters under oxidative stress, demonstrating their sensitivity to temperature-induced volatile changes. In contrast, Cluster 2, composed of Sensors 15, 1, 25, 8, and 23, detected sulfur-containing volatiles such as hydrogen sulfide and ammonia, which increase due to thermal degradation during storage at higher temperatures. This cluster exhibited greater variation across temperatures, with Sensors 15 and 1 showing higher sensitivity and variability at 50 °C, when sulfur compounds formed in greater quantities due to protein breakdown. This suggests that these sensors are less stable at higher temperatures and are more sensitive to thermal degradation products.

These findings highlight how volatile compound formation varies with temperature, and how individual sensors exhibit distinct stability and sensitivity to these shifts. Similar sensor grouping trends have been reported in E-nose applications; for instance, Hong et al. (37) applied a semi-supervised “Cluster-then-Label” model to classify the freshness of cherry tomato juice, demonstrating a reliable prediction of juice quality using chemometric features. Additionally, Hong et al. (38) found that hierarchical clustering remained a robust method for distinguishing between freshness and adulteration levels in tomato juices compared to spectral and partitioning algorithms. While clustering provided insights into how sensor responses group by compound type and thermal behavior, feature importance analysis further quantified the individual contribution of each sensor to temperature classification accuracy.

#### 3.4.3 Feature importance analysis

**Figure 9:**
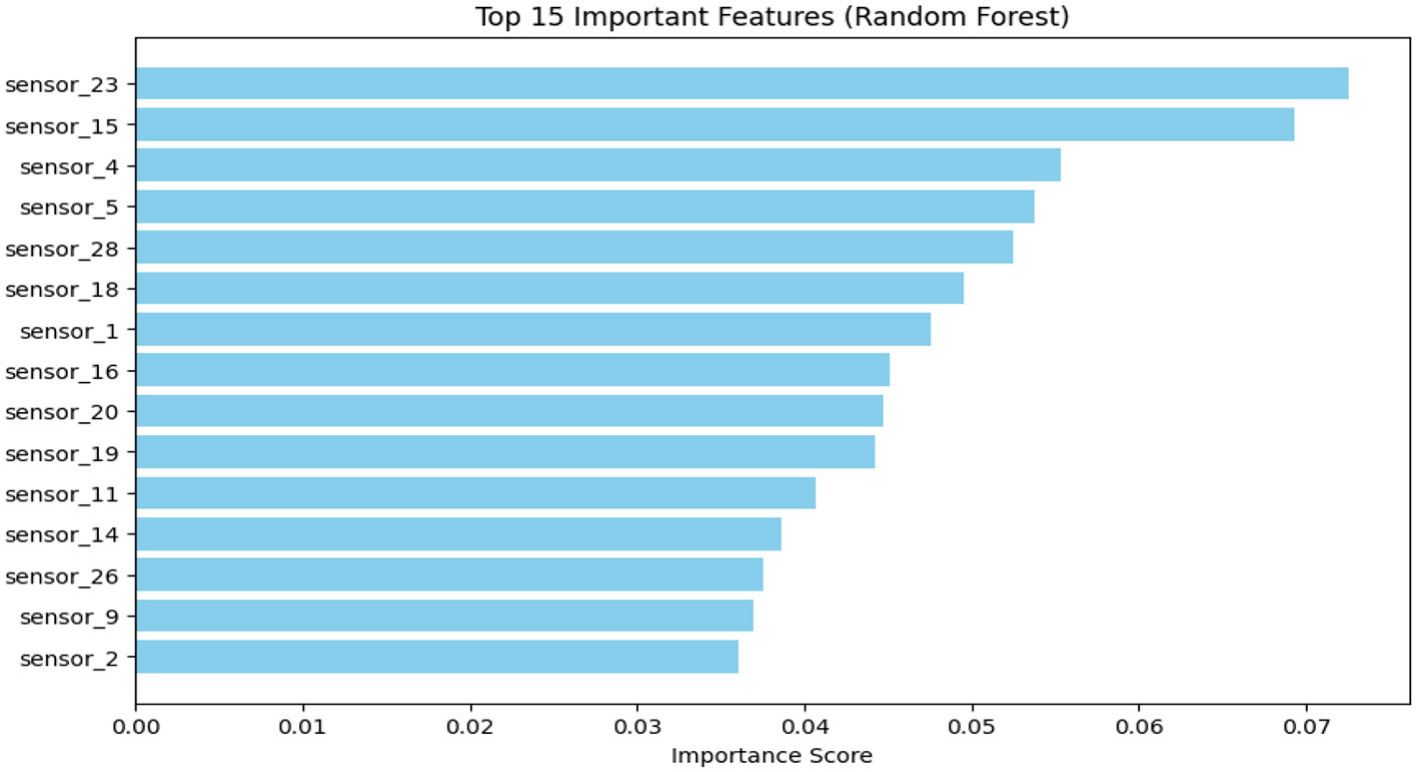
Top 15 E-nose sensors ranked by importance scores in random forest classification of storage temperatures.

The Random Forest feature ranking (Figure 9) showed that sensors responsive to alcohols, esters, and sulfur-containing volatiles were most influential in distinguishing storage temperatures. This pattern is consistent with temperature-dependent changes in volatile composition, in which moderate heating favors the formation of alcohols and esters, while higher temperatures are associated with increased detection of sulfur-related volatiles.

Sensor 4, known for detecting sulfur volatiles such as hydrogen sulfide and ammonia, played a key role in distinguishing between 50 °C samples, where thermal degradation intensified. Similarly, Sensor 28, which is responsive to esters such as ethyl acetate, showed increased activity under high-temperature storage, reflecting enhanced lipid oxidation and esterification.

In contrast, Sensors 15 and 1, which detect alcohols and esters, demonstrated relatively stable responses across RT and 40 °C samples. Their consistent behavior suggests that these sensors are reliable markers of moderate temperature-induced changes in the volatile matrix, without being overwhelmed by high-temperature degradation artifacts.

Overall, the findings suggest that sensors targeting esters and alcohols (Sensors 1, 15, and 28) indicate moderate thermal transformation, while those tuned to sulfur volatiles (e.g., Sensor 4) are critical in capturing the effects of thermal stress. These trends are consistent with previous E-nose studies—Rizzolo et al. (39) reported high feature importance for alcohol- and ester-sensitive sensors in monitoring peach ripening during cold storage, while Hong et al. (37) used similar sensor ranking strategies in tomato juice freshness classification using a Random Forest model.

### 3.5 Thermal effects on phenolic stability and degradation kinetics

TPC was selected for kinetic modeling as a representative marker of phytochemical degradation, whereas antioxidant capacity reflects multiple interacting components and was therefore evaluated descriptively rather than kinetically. Kinetic modeling was conducted for the optimized formulation (Sample F), which demonstrated favorable overall performance across most key sensory, physicochemical, and multivariate quality indicators and was therefore selected for stability modeling.

TPC degradation in the vinegar-based beverage demonstrated a clear temperature-dependent pattern. Over the 2-month storage period, a substantial decline in TPC was observed at elevated temperatures, with the first-order degradation rate constant (k) increasing from approximately 0.85/month at 25 °C to 1.43/month at 50 °C (see Supplementary Table S2). This supports the assumption that TPC degradation followed first-order kinetics under the tested conditions.

**Figure 10:**
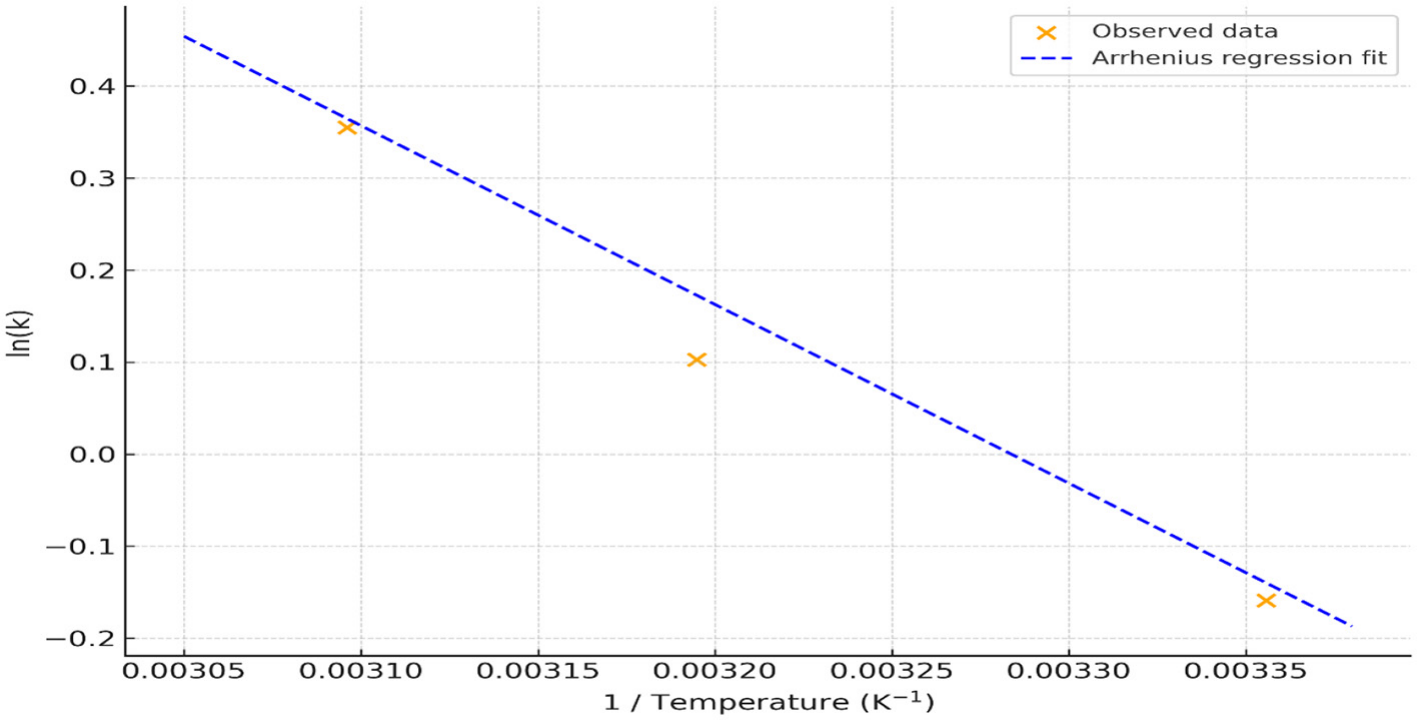
Arrhenius plot of TPC degradation. Points represent observed ln (k) values; the dashed line shows the regression fit (Ea ≈ 16.2 kJ/mol).

The Arrhenius plot (Figure 10), which plots the natural logarithm of the degradation constants (ln k) against the reciprocal of absolute temperature (1/T), shows a strong linear correlation, validating the applicability of the Arrhenius model for describing the thermal degradation behavior of phenolics in this matrix. The calculated activation energy (Ea) was approximately 16.2 kJ/mol, indicating a moderate thermal sensitivity typical of polyphenol-rich systems. However, the Arrhenius-derived predictions should be interpreted as indicative of temperature dependence and comparative degradation behavior rather than absolute shelf-life forecasts, as independent validation at additional storage temperatures was beyond the scope of the present study.

The observed TPC decline with increasing temperature is consistent with prior reports on thermally processed functional beverages. For example, Shi et al. (40) reported a significant decrease in TPC and antioxidant capacity in date seed beverages with increasing roasting temperatures (180–220 °C), highlighting the thermal instability of phenolic constituents. Similarly, De Beer et al. (41) noted the accelerated degradation of major phenolics in honeybush iced tea powders stored at 40 °C. However, matrix-specific variability exists; for instance, Abduh et al. (42) found that higher brewing temperatures increased extractable TPC in cascara but concurrently reduced antioxidant efficacy.

Collectively, these findings support the conclusion that elevated storage temperatures compromise the stability of phenolics, resulting in a loss of antioxidant potential in plant-based functional beverages. Thus, controlling thermal exposure is crucial for maintaining product quality and retaining bioactivity.

### 3.6 Limitations

A key limitation of this study is that it evaluated physicochemical, phytochemical, sensory, and machine-learning-based quality changes during storage, but microbiological analysis was not performed. Given the known antimicrobial properties of red date vinegar, honey, and goji phytochemicals, future studies should incorporate microbiological assessments to validate safety and shelf life. Additionally, while titratable acidity was screened during preliminary formulation trials and assessed sensorially in the final samples, future research will include quantitative acidity measurements to further characterize product stability. The relatively short storage duration in this study is acknowledged, and extended storage evaluations will be considered in future work.

## 4 Conclusions

Storage temperature significantly influenced the compositional stability and sensory quality of the sun-aged red date vinegar–based beverage. Samples stored at 25 °C retained balanced aroma and sweetness, whereas those stored at 40 °C showed greater fruitiness but greater sensory variability. Elevated storage temperature (50 °C) accelerated the loss of volatile compounds and phenolic constituents, indicating reduced compositional stability under thermal stress. E-nose analysis combined with machine learning successfully identified temperature-responsive volatile markers, demonstrating the value of multivariate analytical tools for monitoring storage-induced quality changes. Overall, the results highlight the importance of temperature control in maintaining compositional and sensory stability in vinegar-based beverages and demonstrate the utility of integrated analytical and data-driven approaches for beverage formulation and shelf-life evaluation. While these findings underscore the importance of temperature control for maintaining product quality, the use of machine learning for real-time monitoring remains an area for further development.

## Data Availability

The original contributions presented in the study are included in the article/Supplementary material, further inquiries can be directed to the corresponding authors.

## References

[1] Khalifa SA El-Shabasy RM Tahir HE Abo-Atya DM Saeed A Abolibda T . Vinegar-the beneficial food additive: production, safety, possibilities, and applications from ancient to modern times. Food Func. (2024) 15:10262–82. doi: 10.1039/D4FO02377C 39327882

[2] Sethy S Sharma K Dagar A Mogra R. Effect of storage on physico-chemical and sensory qualities of commercial fruit beverages. Int J Current Microbiol Appl Sci. (2018) 7:1138–47. doi: 10.20546/ijcmas.2018.710.126

[3] Cantadori E Brugnoli M Centola M Uffredi E Colonello A Gullo M. Date fruits as raw material for vinegar and non-alcoholic fermented beverages. Foods. (2022) 11:1972. doi: 10.3390/foods11131972 35804787 PMC9265875

[4] Ali Z Ayub A Wen Lin Y Anis S Khan I Younas S . Lycium Barbarum's diabetes secrets: a comprehensive review of cellular, molecular, and epigenetic targets with immune modulation and microbiome influence. J Pharmaceutical Anal. (2024) 101130. doi: 10.1016/j.jpha.2024.101130 PMC12151213 40496070

[5] Goh K Ng S Nyam K. The infusion of goji berries and red dates ameliorates the overall qualities of kenaf leaves tea. Int Food Res J. (2021) 28:1216–22. doi: 10.47836/ifrj.28.6.13

[6] Wang W Jia R Hui Y Zhang F Zhang L Liu Y . Utilization of two plant polysaccharides to improve fresh goat milk cheese: texture, rheological properties, and microstructure characterization. J Dairy Sci. (2023) 106:3900–17. doi: 10.3168/jds.2022-22195 37080791

[7] Cao H Saroglu O Karadag A Diaconeasa Z Zoccatelli G Conte-Junior CA . Available technologies on improving the stability of polyphenols in food processing. Food Front. (2021) 2:109–39. doi: 10.1002/fft2.65

[8] Marsol-Vall A Laaksonen O Yang B. Effects of processing and storage conditions on volatile composition and odor characteristics of blackcurrant (*Ribes nigrum*) juices. Food Chem. (2019) 293:151–60. doi: 10.1016/j.foodchem.2019.04.076 31151596

[9] Malfeito-Ferreira M. Fine wine flavour perception and appreciation: blending neuronal processes, tasting methods and expertise. Trend Food Sci Technol. (2021) 115:332–46. doi: 10.1016/j.tifs.2021.06.053

[10] Putri LA Rahman I Puspita M Hidayat SN Dharmawan AB Rianjanu A . Rapid analysis of meat floss origin using a supervised machine learning-based electronic nose towards food authentication. NPJ Sci Food. (2023) 7:31. doi: 10.1038/s41538-023-00205-2 37328497 PMC10275922

[11] Ali Z Rafique H Tahir RA Saeed T Rasheed MA Khan I . Clinical and computational exploration of red date fruit vinegar: synergistic effects on cardiovascular and type 2 diabetes pathways. Front Nutr. (2025) 12:1557733. doi: 10.3389/fnut.2025.1557733 40727700 PMC12302754

[12] Ali Z Brottier ZR Al-Khayri JM Tahir RA Al-Dalali S Al-Dossary O . Sun-aged red date vinegar-based beverage: integrated analysis of fermentation, sensory, volatile, and bioactive properties. Food Chem: X. (2025) 103023. doi: 10.1016/j.fochx.2025.103023 41017929 PMC12475855

[13] Pauline M Alexandre O Andoseh BK Abeline MTS Agatha T. Production technique and sensory evaluation of traditional alcoholic beverage based maize and banana. Int J Gastr Food Sci. (2017) 10:11–5. doi: 10.1016/j.ijgfs.2017.09.003

[14] Singleton VL Orthofer R Lamuela-Raventós RM. [14] Analysis of total phenols and other oxidation substrates and antioxidants by means of folin-ciocalteu reagent. In *Methods in Enzymology*. (1999). Amsterdam: Elsevier. p. 152–78. doi: 10.1016/S0076-6879(99)99017-1

[15] Chen CW Ho CT. Antioxidant properties of polyphenols extracted from green and black teas. J Food Lipids. (1995) 2:35–46. doi: 10.1111/j.1745-4522.1995.tb00028.x

[16] Re R Pellegrini N Proteggente A Pannala A Yang M Rice-Evans C. Antioxidant activity applying an improved ABTS radical cation decolorization assay. Free Rad Biol Med. (1999) 26:1231–7. doi: 10.1016/S0891-5849(98)00315-3 10381194

[17] World Medical Association. World medical association declaration of Helsinki: ethical principles for medical research involving human subjects. JAMA. 310:2191–4. doi: 10.1001/jama.2013.281053 24141714

[18] Shariati M Touranlou FA Rezaie M. Sensory evaluation methods for food products targeting different age groups: a review. Food Res Int. (2025) 221:117608. doi: 10.1016/j.foodres.2025.117608 41185354

[19] Tobin R Moane S Larkin T. Sensory evaluation of organic and conventional fruits and vegetables available to Irish consumers. Int J Food Sci Technol. (2013) 48:157–62. doi: 10.1111/j.1365-2621.2012.03172.x

[20] Mamat M Samad SA Hannan MA. An electronic nose for reliable measurement and correct classification of beverages. Sensors. (2011) 11:6435–53. doi: 10.3390/s110606435 22163964 PMC3231460

[21] Miti M. Kinetic and thermodynamic characteristics of thermal degradation of anthocyanins from strawberry and blueberry commercial juices. Chem Chem Naissensi. (2020) 3:46–63. doi: 10.46793/ChemN3.2.046M

[22] Frangopoulos T Koliouskas A Petridis D. The effect of accelerated storage temperature conditions on the shelf life of pasteurized orange juice based on microbiological, physicochemical, and color attributes. Appl Sci. (2024) 14:10870. doi: 10.3390/app142310870

[23] Mgaya-Kilima B Remberg SF Chove BE Wicklund T. Influence of storage temperature and time on the physicochemical and bioactive properties of roselle-fruit juice blends in plastic bottle. Food Sci Nutr. (2014) 2:181–91. doi: 10.1002/fsn3.97 24804077 PMC3959965

[24] Singh SK Sharma M. Review on biochemical changes associated with storage of fruit juice. Int J Cur Microbiol Appl Sci. (2017) 6:236–45. doi: 10.20546/ijcmas.2017.608.032

[25] Long X Li R Gu J Zhang L Guo S Fan Y . Changes in phenolic compounds of *Phyllanthus emblica* juice during different storage temperature and pH conditions. J Food Sci. (2024) 89:4312–30. doi: 10.1111/1750-3841.17129 38865254

[26] Aaby K Amundsen MR. The stability of phenolic compounds and the colour of lingonberry juice with the addition of different sweeteners during thermal treatment and storage. Heliyon. (2023) 9:e15959. doi: 10.1016/j.heliyon.2023.e15959 37215818 PMC10192756

[27] Mrázková M Sumczynski D Orsavová J. Influence of storage conditions on stability of phenolic compounds and antioxidant activity values in nutraceutical mixtures with edible flowers as new dietary supplements. Antioxidants. (2023) 12:962. doi: 10.3390/antiox12040962 37107337 PMC10135932

[28] Silva CNd Carmo JRd Nunes BV Demoliner F Souza VR Bastos SC. Synergistic effect of thermosonication on the stability of bioactive compounds and antioxidant activity of blackberry juice. FoodS. (2025) 14:901. doi: 10.3390/foods14050901 40077604 PMC11898737

[29] Maulida ID Al Marsam MR Purnama I Mutamima A. A novel beverage with functional potential incorporating cascara (*Coffea arabica*), roselle (*Hibiscus sabdariffa*), and red ginger (*Zingiber officinale* Rosc. var rubrum) extracts: chemical properties and sensory evaluation. Discover Food. (2024) 4:94. doi: 10.1007/s44187-024-00180-x

[30] Rezazadeh A Ghasempour Z. Anthocyanin stabilizationfsn in beverages. in Natural Products in Beverages: botany, phytochemistry, pharmacology and processing. Berlin: Springer. (2024) p. 1–36. doi: 10.1007/978-3-031-04195-2_178-1

[31] Laaksonen O Mäkilä L Jokinen M Metz T Kallio H Yang B. Impact of storage on sensory quality of blackcurrant juices prepared with or without enzymatic treatment at industrial scale. Eur Food Res Technol. (2020) 246:2611–20. doi: 10.1007/s00217-020-03601-0

[32] Kishore K Pathak K Shukla R Bharali R. Effect of storage temperature on physico-chemical and sensory attributes of purple passion fruit (*Passiflora edulis* Sims). J Food Sci Technol. (2011) 48:484–8. doi: 10.1007/s13197-010-0189-8 23572775 PMC3551175

[33] Zia H Slatnar A Košmerl T Korošec M. A review study on the effects of thermal and non-thermal processing techniques on the sensory properties of fruit juices and beverages. Front Food Sci Technol. (2024) 4:1405384. doi: 10.3389/frfst.2024.1405384

[34] Fei C Xue Q Li W Xu Y Mou L Li W . Variations in volatile flavour compounds in Crataegi fructus roasting revealed by E-nose and HS-GC-MS. Front Nutr. (2023) 9:1035623. doi: 10.3389/fnut.2022.1035623 36761989 PMC9905410

[35] Li Q Gu Y Wang N-F. Application of random forest classifier by means of a QCM-based e-nose in the identification of Chinese liquor flavors. IEEE Sens J. (2017) 17:1788–94. doi: 10.1109/JSEN.2017.2657653

[36] Ni W Wang T Wu Y Chen X Cai W Zeng M . Classification and concentration predictions of volatile organic compounds using an electronic nose based on XGBoost-random forest algorithms. IEEE Sens J. (2023) 24:671–8. doi: 10.1109/JSEN.2023.3304355

[37] Hong X Wang J Qi G. E-nose combined with chemometrics to trace tomato-juice quality. J Food Eng. (2015) 149:3843. doi: 10.1016/j.jfoodeng.2014.10.003

[38] Hong X Wang J Qi G. Comparison of spectral clustering, K-clustering, and hierarchical clustering on e-nose datasets: application to the recognition of material freshness, adulteration levels, and pretreatment approaches for tomato juices. Chemometr Intelligent Lab Syst. (2014) 133:17–24. doi: 10.1016/j.chemolab.2014.01.017

[39] Rizzolo A Bianchi G Vanoli M Lurie S Spinelli L Torricelli A. Electronic nose to detect volatile compound profile and quality changes in ‘Spring Belle' peach (*Prunus persica* L.) during cold storage in relation to fruit optical properties measured by time-resolved reflectance spectroscopy. J Agricul Food Chem. (2013) 61:1671–85. doi: 10.1021/jf302808g 23020286

[40] Shi L Ghafoor K Viejo CG Jara SAF Ahmadi F Suleria HA. Impact of roasting temperature on antioxidant activities and characterization of polyphenols in date seed beverages from different cultivars. J Food Sci. (2025) 90:e70242. doi: 10.1111/1750-3841.70242 40331789 PMC12057541

[41] De Beer D Pauck CE Aucamp M Liebenberg W Stieger N van der Rijst M . Phenolic and physicochemical stability of a functional beverage powder mixture during storage: effect of the microencapsulant inulin and food ingredients. J Sci Food Agric. (2018) 98:2925–34. doi: 10.1002/jsfa.8787 29168179

[42] Abduh MY Nofitasari D Rahmawati A Eryanti AY Rosmiati M. Effects of brewing conditions on total phenolic content, antioxidant activity and sensory properties of cascara. Food Chem Adv. (2023) 2:100183. doi: 10.1016/j.focha.2023.100183

