# Supplementary material for: Storage temperature and quality dynamics of sun-aged red date vinegar beverage
Source: Front Nutr. 2026 May 18;13:1839712. doi: 10.3389/fnut.2026.1839712 (PMC13222999; doi:10.3389/fnut.2026.1839712)
Supplement: Supplementary file 1 [file Table_1.docx]

# Supplementary Table S1. Description of Electronic Nose (E-nose) Sensors and Their Detection Ranges

| **Sensor** | **Target Compound Class** | **Detection Range (ppm)** |
| --- | --- | --- |
| S1 | Alkanes, fumes | 200–10,000 |
| S2 | Alcohols, aldehydes | 1–1,000 |
| S3 | Short-chain alkanes, ozone | 10–1,000 |
| S4 | Sulphide, hydrogen | 1–200 |
| S5 | Sulphide, ammoniates | 5–500 |
| S6 | Ammonia, benzene ring compounds, aldehydes, aromatics | 5–500 |
| S7 | Short-chain alkanes, natural gas | 300–10,000 |
| S8 | Short-chain alkanes | 300–10,000 |
| S9 | Short-chain alkanes | 0–500 |
| S10 | Hydrogen | 100–1,000 |
| S11 | Biogas, short-chain alkanes | 300–10,000 |
| S12 | Allyl sulphide | 300–10,000 |
| S13 | Ketones, alcohols | 300–10,000 |
| S14 | Methane | 300–10,000 |
| S15 | Combustible gas | 1–30 |
| S16 | Volatile organic compounds, unusual | 500–10,000 |
| S17 | Odors, butane, LPG, methane | 500–10,000 |
| S18 | Natural gas, propane, butane | 500–10,000 |
| S19 | Short-chain alkanes, methane | 100–1,000 |
| S20 | Volatile organic compounds, alcohols | 1–50 |
| S21 | Methanol, amines, sulfur-smelling gas | 1–200 |
| S22 | Aliphatic hydrocarbons, alicyclics | 1–100 |
| S23 | Halogenated hydrocarbons, air | 1–30 |
| S24 | Polluted air: alkanes, olefins, hydrogen | 30–1,000 |
| S25 | Methane, propane | 300–10,000 |
| S26 | Organic acid esters | 500–10,000 |
| S27 | Mushroom alkenes, alcohol | 1–30 |
| S28 | Refrigerant gases | 100–10,000 |
