# Supplementary material for: Storage temperature and quality dynamics of sun-aged red date vinegar beverage
Source: Front Nutr. 2026 May 18;13:1839712. doi: 10.3389/fnut.2026.1839712 (PMC13222999; doi:10.3389/fnut.2026.1839712)
Supplement: Supplementary file 2 [file Table_2.docx]

**Supplementary Table S2. Raw and calculated data for thermal degradation kinetics of total phenolic content (TPC) during storage.**

| **Parameter** | **25 °C / 298 K** | **40 °C / 313 K** | **50 °C / 323 K** |
| --- | --- | --- | --- |
| Fresh TPC Replicate 1 | 3.35 | 3.35 | 3.35 |
| Fresh TPC Replicate 2 | 3.37 | 3.36 | 3.35 |
| Fresh TPC Replicate 3 | 3.36 | 3.38 | 3.36 |
| TPC After 2 Months Replicate 1 | 0.62 | 0.37 | 0.20 |
| TPC After 2 Months Replicate 2 | 0.60 | 0.37 | 0.18 |
| TPC After 2 Months Replicate 3 | 0.61 | 0.36 | 0.20 |
| TPC 2M Mean | 0.61 | 0.37 | 0.19 |
| TPC 2M SD | 0.01 | 0.00 | 0.01 |
| k (month⁻¹) | 0.8531 | 1.1081 | 1.4266 |
| ln(k) | –0.1589 | +0.1027 | +0.3553 |
| 1/T (K⁻¹) | 0.0033557 | 0.0031949 | 0.0030960 |

All TPC values are expressed in arbitrary units. k represents the first-order degradation rate constant calculated as k=ln(C0/Ct)/t. Activation energy was derived from the Arrhenius regression of ln(k) versus 1/T.
